# An Energy-Efficient and Blockchain-Integrated Software Defined Network for the Industrial Internet of Things

**DOI:** 10.3390/s22207917

**Published:** 2022-10-18

**Authors:** Sasikumar Asaithambi, Logesh Ravi, Hossam Kotb, Ahmad H. Milyani, Abdullah Ahmed Azhari, Senthilkumar Nallusamy, Vijayakumar Varadarajan, Subramaniyaswamy Vairavasundaram

**Affiliations:** 1 Department of Electronics and Communication Engineering, Vel Tech Rangarajan Dr. Sagunthala R&D Institute of Science and Technology, Avadi, Chennai 600062, Tamil Nadu, India; 2 SENSE, Vellore Institute of Technology, Chennai 600127, Tamil Nadu, India; 3 Data Engineering Research Group (DERG–SENSE), Vellore Institute of Technology, Chennai 600127, Tamil Nadu, India; 4 Department of Electrical Power and Machines, Faculty of Engineering, Alexandria University, Alexandria 21544, Egypt; 5 Department of Electrical and Computer Engineering, King Abdulaziz University, Jeddah 21589, Saudi Arabia; 6 Center of Research Excellence in Renewable Energy and Power Systems, King Abdulaziz University, Jeddah 21589, Saudi Arabia; 7 The Applied College, King Abdulaziz University, Jeddah 21589, Saudi Arabia; 8 Department of Electronics and Communication Engineering, M.Kumarasamy College of Engineering, Karur 639113, Tamil Nadu, India; 9 School of Computer Science and Engineering, University of New South Wales, Sydney 2052, Australia; 10 Ajeenkya DY Patil University, Pune 412105, Maharashtra, India; 11 Swiss School of Business Management, SSBM, 1213 Geneva, Switzerland; 12 School of Computing, SASTRA Deemed University, Thanjavur 613401, Tamil Nadu, India

**Keywords:** software-defined networking, blockchain, industrial internet of things, cluster head selection, energy model

## Abstract

The number of unsecured and portable Internet of Things (IoT) devices in the smart industry is growing exponentially. A diversity of centralized and distributed platforms have been implemented to defend against security attacks; however, these platforms are insecure because of their low storage capacities, high power utilization, single node failure, underutilized resources, and high end-to-end delay. Blockchain and Software-Defined Networking (SDN) are growing technologies to create a secure system and to ensure safe network connectivity. Blockchain technology offers a strong and trustworthy foundation to deal with threats and problems, including safety, privacy, adaptability, scalability, and security. However, the integration of blockchain with SDN is still in the implementation phase, which provides an efficient resource allocation and reduced latency that can overcome the issues of industrial IoT networks. We propose an energy-efficient blockchain-integrated software-defined networking architecture for Industrial IoT (IIoT) to overcome these challenges. We present a framework for implementing decentralized blockchain integrated with SDN for IIoT applications to achieve efficient energy utilization and cluster-head selection. Additionally, the blockchain-enabled distributed ledger ensures data consistency throughout the SDN controller network and keeps a record of the nodes enforced in the controller. The simulation result shows that the proposed model provides the best energy consumption, end-to-end latency, and overall throughput compared to the existing works.

## 1. Introduction

The advancement of modern technologies has made the world smarter. Numerous things, which were previously impossible, are now possible for people. The Internet of Things (IoT) is improving our living spaces. To help human activities, innumerable sensors related to various applications are connected together. The IoT components gather data from the physical sensors and can transmit data to the computing system to convert it into an important decision. In the IoT space, the concept of employing RFID is to identify sensors among the thousands of nodes that are accurate and successful [1]. According to some studies, there may be 75 billion gadgets worldwide by 2025 [2]. Because of this, it’s become more likely to be targeted by internet attackers and extremely challenging to manage such massive amounts of information on time.

Industrial 4.0 are integrated with IoT devices, including robots, electronics, and humans, to enable smart manufacturing activities for real-time application [3]. Pervasive edge computing (PEC) is a type of edge processing technology which enables IoT devices, such as portable systems, cell phones, and automation machinery locally, to generate, share, and process data between peer edge platforms [4]. The PEC method has some safety and confidentiality issues and poor networking efficiency. The PEC design in the IIoT context typically comprises three layers: the cloud, the ubiquitous edge, and the IIoT device. The PEC faces several obstacles.

**Figure 1:**
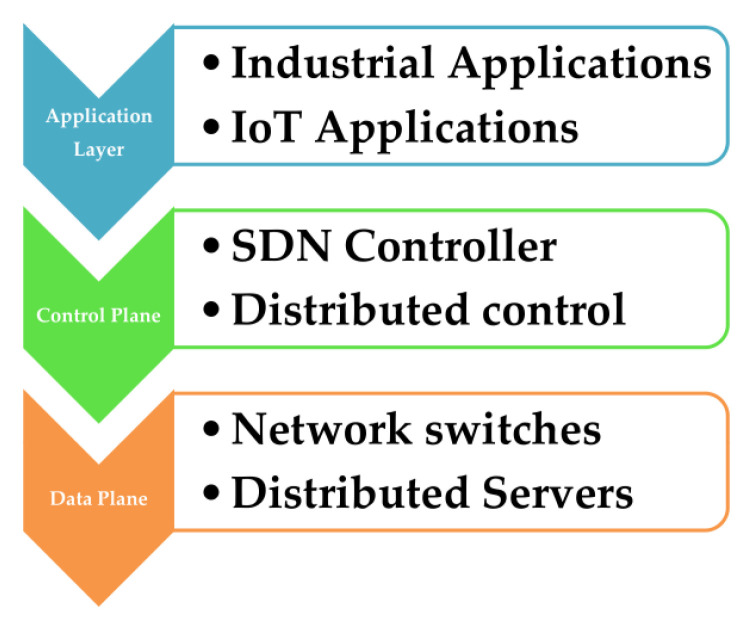
Basic Data flow Structure of SDN.

An advanced intelligent network is developed to supervise the hardware data plane and to individually monitor the logic-plane [5]. The IoT-based SDN network centrally controls various smart devices, such as sensing devices linked to the network. SDN infrastructure substantially supports the smart integration of IoT networks. Customizable SDN network architecture can resolve the adaptability, security, configuration management, device management, and other restrictions of conventional IoT networks [6]. The number of devices linked to IoT networks in smart cities is increasing the volume of traffic on those networks. Figure 1 shows the basic SDN architecture to control the massive data flow of smart city IoT networks. In smart city systems, several SDN controllers handle the data flow of various IoT networks. The IoT systems and SDN integrated architecture can improve the user data flow stream’s ability in the network.

End-to-end IoT devices frequently demand smart city apps and services at various locations. The demand for new software solutions surged due to the IoT device market’s exponential rise. The smart city may utilize many IoT infrastructures, including machine learning, wireless services, video streaming, autonomous traffic control, smart monitoring, digital user interactions, and safety. These applications have strict requirements for greater quality of service (QoS), reduced delay, adaptive control, and control, which traditional network environments cannot provide. To satisfy security needs, the IoT should adopt new architecture models, such as SDN, which can be the potential solution for IoT-enabled networks. Regulating the logic-plane and monitoring the hardware data-plane are independently controlled by SDN, allowing the network to be centrally managed and made programmable to support the network infrastructure for multiple application services. The combination of SDN with IoT networks may improve system efficiency for smart industry and make networks more adaptable and accessible.

At the end of 2023, Machine to Machine (M2M) interaction will account for half of all smart components and interactions worldwide, according to the most recent Cisco Internet Report [7]. In particular, it is predicted that home automation devices would account for 48% of M2M interactions. In comparison, connected vehicle services will experience a 30% compound annual growth rate throughout the projected period of 2018–2023. It is then getting harder to manage these enormous numbers of devices and the network that connects them since the IoT ecosystem is more complex and diverse than a typical processing unit, which worsens the IoT’s significant security challenges.

Blockchain is one of the most sophisticated and well-developed techniques to protect internet information exchange, so it is typically referred to as decentralized network architecture. A distributed network [8] receives huge attention for implementing security systems and is suitable for the decentralization configuration of the IoT platform. Additionally, blockchain offers a decentralized framework that stores digital records, transmits them over the system, and never permits third parties to change the information. Since most applications need a distributed database, where information may be concurrently stored and accessed over the Internet, on request with reliability, which is managed by a cloud computing platform, the combination of a cloud network with IoT sensors gives a bigger and more scalable storage capacity and the connectivity required to quickly communicate information among the devices and derive meaning from it. Furthermore, the IoT paradigm connects various physical devices through the Internet, and the number of these connections is growing daily.

A centralized SDN controller is employed to monitor the entire system during the earliest stages of the implementation of SDN-integrated IoT systems while also addressing certain IoT network optimizations. On the other hand, several controllers have been investigated with the primary objective of reducing packet loss. Integrating SDN and blockchain into IoT systems enables enhanced privacy and security control [9,10]. To ensure network transmission reliability, the devices must be monitored properly to avoid node failures. Security mechanisms that enable the manufacturer to authorize IoT data access in the network have been implemented in [11], with blockchain as the main objective for security. More recently, time slot management-aware clustering techniques have been introduced. The clustering techniques enable the most effective and efficient usage of sensors or IoT gadgets. Other blockchain-related parameters need to be controlled while using these cluster-based optimizations. Researchers continue to struggle with the cluster head selection model. To manage and monitor the network, the SDN integrated IoT system has been implemented in many works [12] and proposed various network architectures.

Based on these factors, managing the capabilities of the IoT platform is essential to addressing the major issues related to SDN integration, such as confidentiality and protection [13]. There are still several issues to be resolved to facilitate blockchain in the SDN environment to create the IoT community. To achieve this, we suggest a layered hierarchical design in the present study for effectively tackling various resource allocation concerns in a decentralized smart contract integrated SDN-IoT framework. In the IoT layer, we also created a contemporary cluster head selection technique that is quicker and uses less energy than the baseline. Additionally, we use a blockchain-enabled flow rules record to ensure the decentralized controller cluster’s reliability.

### Security Challenges and Solutions with SDN

To address open and dependable security issues, we propose the blockchain-enabled SDN integrated IoT framework, a decentralized security system for industrial applications. Its core concept is that access SDN data encrypted by blockchain networks are used to approve IoT nodes and that these devices should be distributed as a decentralized system. The blockchain can only provide data security and be used to access SDN-IoT resources appropriately. While the blockchain enables trustworthy data transfer and records between diverse and untrusted SDN domains, the proposed framework provides flexible and fine-grained authorization management of IoT networks. We specifically incorporate blockchain capabilities into full SDN-IoT nodes. The authentication process features on controllers are implemented using the smart contract in an intuitive and non-deniable way. The transaction acts as the network interface system to transfer data across network entities. We developed blockchain-based initialization and SDN controller-based updating techniques to track the times a node is used to transfer data in the network.

We develop the SDN control architecture that separates permission from the call process of blockchain to reduce the latency and complexity issues associated with the IoT network. The SDN architecture has low access latency since it requires authorization before calling IoT resources. To address this issue, we present a small enhancement to the blockchain to cut down on the quantity of data that needs to be managed. Finally, we validate the proposed architecture efficacy against the baseline SDN network via various performance analyses. To assess the performance of the design, we also implement it using the simulation environment. The outcomes demonstrate that the proposed method can offer efficient security with reasonable overheads.

The following summarizes our significant contributions.

- This article presents a unique security block control method based on blockchain for use in SDN-integrated IoT networks. It can record all interactions between SDN and IoT resources and facilitate proper framework security in diverse and untrusted industrial applications.
- By integrating blockchain and SDN, we enhance trustworthiness in the framework. For SDN integrated IoT architecture, dedicated energy-aware cluster head algorithms are created to meet IoT devices management needs for the decentralized, dependable, and adaptable access control architecture.
- Since blockchain technology is still in its infancy, this work’s key contribution addresses how to utilize it while minimizing its energy fully. To decrease the request latency of SDN, we describe the access control framework here that separates data and control plane.

The remainder of this work is structured as follows: A complete review of relevant work is provided in Section 2. Section 3 provides complete detail on the proposed system architecture and cluster head selection method. Section 4 presents the proposed framework’s simulation setup and performance analysis compared with existing models. The conclusion is given in Section 5.

## 2. Background and Related Work

The following sub-sections discuss the relevant works integrating SDN with IoT networks and SDN with blockchain techniques. Additionally, this section describes the basic concept and latest implementations of SDN and IoT networks. For a clear understanding, the related work is presented in subsections.

### 2.1. Basic of Internet of Things

The definition of (IoT) is a setting where physical items are connected to the network in a way that makes them active players in business operations. These things can range from sensors to household and medical supplies to network gadgets. These items or gadgets have been given a special address linked to the Internet. The IoT consists of a heterogeneous spectrum of devices that can be wired or wireless, use a variety of protocols, and be a part of numerous contexts and networks [14]. By 2021, it is predicted that more than 46 billion IoT devices will be in operation, according to the latest Juniper research [15]. IoT becomes a key element in any emerging computer and networking architecture.

The IoT revolution is rapidly growing, leading to significant automation advancements and financial benefits. Numerous obstacles are presented by the diverse nature of IoT devices and the IoT environment. It is challenging to find universal solutions for all of these things because they are created to satisfy the needs of particular user goals. Some serious cyber security risks that antivirus program scans are mentioned concerning the security problems faced in the IoT environment. One of the first bits of the virus was found in IoT, Ubuntu, and Darlloz, reported by the Symantec organization. Real-time IoT device detection systems could result in high overhead costs. This article introduces software-defined networks (SDN) to emphasize network flexibility. The data plane is only useful to handle data transfer because the control plane is segregated from it. All network-wide decision-making is the responsibility of the control plane. We created a system that can be used with various IoT devices using an SDN-based approach. Therefore, SDN ensures that IoTs will have a bright future in their practicable, real-world applications.

### 2.2. Architecture of Software-Defined Network

The most potential network framework for the future is software-defined networking. Implementation, management, and the data plane, along with associated north and south-bound programs, comprise the three SDN levels. By network optimization, the SDN concept has reduced network services to supplier architecture [16]. The development of SDNs as an emerging network architecture result from the segregation of the control plane and data plane. The innovative and exciting SDN architecture has moved the complete control mechanism to a centralized control plane. As a result, the knowledge and central power of SDNs are what give them their strength and capabilities.

The south-bound interface is used by SDN architecture to collect data from network devices. The most crucial communication in the south bound based SDN architecture is facilitating communication between the controller and network devices [17]. OpenFlow is a standardized networking connection developed by the Open Networking Foundation (ONF) and distinguished by SDN architecture’s data and control planes, which are among the most significant south-bound technologies. This design allows immediate access as well as management of the forward plane components, including routers and switches, by utilizing direct links to modify the forwarding devices [18].

OpenFlow is the most popular protocol in the south-bound interface, where the SDN controller talks to the switch. Other suggestions are NOS, RYU, and P4. The NOS framework supports various open applications, including Floodlight, POX, etc. The SDN framework can use the north-bound interface of the NOS to control the networks [19]. The switch remains connected with the controller with the help of the OpenFlow protocol, which is used to allow packets to be transferred between switches and controllers. OpenFlow includes a routing table and activities that instruct the switch on how and where to handle these streams and channels. All regulations and choices are governed by a controller, who can see the entire system. There are some valid worries concerning the scalability of SDN, although these challenges are not exclusive to SDN technology. The typical control protocol design faces the same difficulties. As a result, rather than stressing SDN, we should be concerned about the scalability problems in conventional networks. Control planes must be implemented with the lowest degree of stability to maintain the flexibility of SDN.

The switching components in a conventional data center may grow quickly. The central controller will be overloaded by the high rate of change in the control signals produced in the network. Installing restrictions on the switches is one approach to solve this issue since it prevents control queries from entering the control plane. The massive quantity of control channel traffic places heavy stress on SDN because it may force control signals to be delayed further. Programmable network abstraction based on SDN is crucial to support various services in cloud architecture. The IoT provides a massive quantity of traffic that can be handled by a smart system at the edge, due to the dynamic deployment of visualized security capabilities.

However, SDN is essential for networks with dynamic configuration and offers new connectivity guidelines as needed. It has been established that the SDN is a potent and adaptable facilitator for network solutions. Additionally, SDN-based systems offer scalability, dynamism, adaptability, and central control, all of which are crucial for improving control decisions. SDN’s control plane can change much functionality because it is totally configurable. 

### 2.3. SDN Integrated IoT Network’s

**Figure 2:**
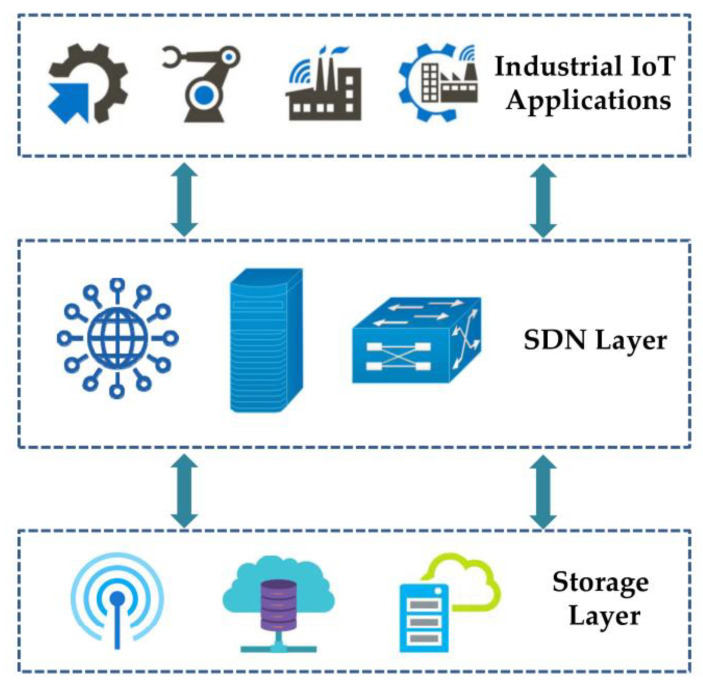
A basic structure of SDN integrated IoT devices.

A Manufacturer’s Use Description (MUD) approach for the network channels presents the official security policies, data protection, and access controls. The MUD is utilized in the SDN platform to locate components, data and resources quickly. MUD also used the blockchain security model to transfer data with the aid of IoT devices via the Hyperledger platform. The security architecture for the dynamic and on-demand administration for virtualized identification, authority, and accountability in SDN-enabled IoT networks was additionally provided by [20,21]. The authors accomplished efficient IoT device bootstrap and fine-grained administration of their network access control. On the other hand, ref [22] provided a revolutionary integration of cloud computing, IoT, and SDN that led to the creation of the CENSOR framework, which is used to offer a safe foundation in the context of IoT. The basic architecture of SDN integrated IoT devices for industrial application is shown in Figure 2.

CENSOR has an SDN-based, cloud-enabled, and dependable IoT network model. The model also identified several obstacles and potential dangers that need to be solved which include enhanced protection, security against Distributed Denial of Service (DDoS) attacks and selecting proper routing algorithms. In [23], the authors suggested a distributed control cluster to manage an SDN’s size, fault-tolerance, compatibility, and dependability challenges. The authors further assert that their solution enhances the device’s performance by achieving proper processor use. Middlebox-Guard (M-G), an SDN-based data transmission security paradigm for fending off various assaults and enhancing system reliability, was created by [24] with a specific focus on IoT application security.

The energy-aware cluster’s head selection algorithm addresses the deployment of IoT devices to a set of enhanced security protocols [25]. The coverage needs of switch node limitations are subsequently satisfied using SDN resource control techniques. The SDN controller separates the data plane and the control plane to enhance security in IoT devices. Hence the integration of SDN with IoT devices will enhance the data flow model between nodes.

### 2.4. SDN-IoT with Blockchain Technology

The “DistBlockSDN” model for smart cities was presented by Rahman et al. in [26]. Authors are provided with a CHS method for sensor data collection with minimal energy loss. The performance of several factors, including throughput and packet arrival rate, was also extensively assessed by the authors by developing blockchain technology. In a different study, Sharma et al. [27] suggested an effective cloud infrastructure to boost security using blockchain technology. However, the presented architecture with a decentralized cloud infrastructure offers secure, low-cost access to the data centers. Additionally, they employed some performance indicators to assess their proposition.

In [28], the authors review the implications of blockchain for cloud-based IoT. The authors covered the requirement for blockchain before its deployment in IoT. In [29], the authors identified blockchain as a potential solution after assessing the security risks, obstacles, effectiveness, and viability of IoT-based applications. In addition, for privacy and security reasons, Ammi et al. [30] examined the key features and functionalities of the smart home based on blockchain for IoT. They employed a local, private blockchain that maintains a time-ordered transaction record for each level of the smart house and offers safe access management for IoT devices.

Finally, most of the research work discussed the security and privacy issues in the IoT applications. Recent researchers are also interested in energy use, and they worried about sustainability. For the IoTs, we have created a distributed approach. The remaining security issues are resolved using blockchain and SDN with the multi-controller network. For energy efficient applications, we proposed a CHS method to reduce the power consumption of IoT devices.

## 3. Proposed Blockchain Enabled SDN Integrated IoT Based Distributed Framework for Industrial Applications

**Figure 3:**
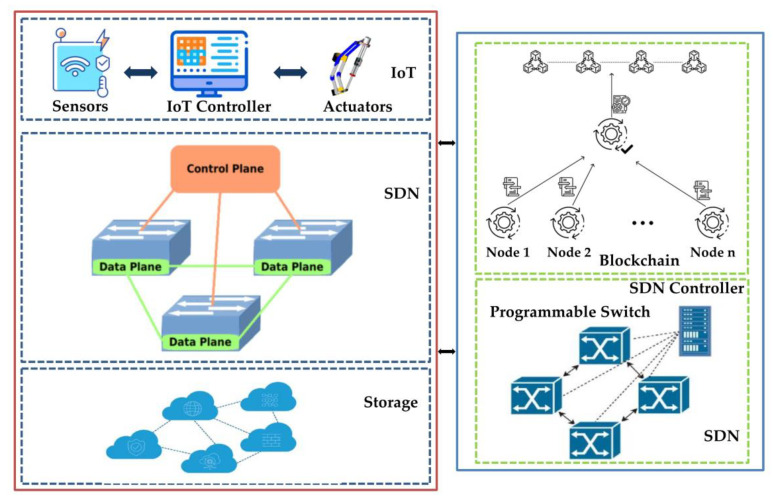
The proposed framework of blockchain enabled SDN-IoT network.

Taking the above security issues and network model, we propose a decentralized blockchain-integrated SDN architecture for IoT applications. The proposed dataflow starts with the perception layer, where users get data from the IoT device. The end layer then generates a report based on the information produced by the perception layer and also analyses the information effectively. Additionally, the cloud layer is utilized by cloud service providers (CSPs) to deliver device activities and store the needed data effectively. Finally, the overall architecture performance of each layer is completely dependent on the blockchain and SDN technologies. A framework of proposed blockchain-enabled SDN as an integrated IoT device for industrial application is illustrated in Figure 3.

The proposed SDN perception layers are divided into several parts to describe our architecture. A cluster head selection (CHS) algorithm is implemented with reduced energy utilization in the perception layer environment. We first present an energy-aware CHS algorithm. The data layer and the control layer are two types of layers that have been created for the SDN cloud architecture. We also implement the simulation environment for SDN-based IoT architecture for transmitting unprocessed data over the data layer of the standard gateway protocol for industrial applications. Additionally, we employ the energy-aware network, which offers physical specification of SDN and efficiently reduces energy usage. The physical specifications include digital network scalability, bandwidth allocation, and energy efficiency.

In the proposed architecture, we have used a FloodLight based controller unit to transmit the valid data to the to the control layer in the SDN framework. The proposed SDN environment implemented using an OpenFlow routing mechanism. Furthermore, SDN controllers guarantee that all data transmitted to the control layer is valid. In addition to that, we integrate the blockchain mechanism with a distributed ledger for performing the six types of transaction between blocks using the network protocol. The six types of transaction requests—release, decrypt, consume, update, and revoke—are created to assist efficient data management. These transactions ensure security and privacy to the SDN framework more confidentially. Finally, the block data are available in the distributed ledger. If the SDN controller receives valid data, then the data are transmitted to the data layer, as depicted in Figure 3. Once the secure data transaction is completed through blockchain technology, cloud data are fed to the IIoT devices, which include smart manufacturing, healthcare, aerospace, and smart traffic applications.

### 3.1. Energy-Aware Cluster Head Selection Method

In the proposed framework, IoT devices can transfer data to the SDN controller through common network gateways. The controller’s job is to filter the usable information from connected devices. The developed architecture will ensure privacy and security to the IoT device, which will be transferred to the cloud layer. The major limitation to IoT device communication performance is the selection of the cluster head in the network. The following part discusses the proposed energy-aware cluster head selection algorithm for SDN-enabled IoT applications. The selection of cluster heads is the most important part of the proposed architecture. Since the computing energy is mostly dependent on the power utilization of node selection [31], to increase the life span time of the node, the heads are randomly distributed within the network. The initial process of CHS starts with ordering each node based on their energy. As formerly emphasized, the clustering stage is only carried out once before cluster head selections are carried out, and only one head is changed per round.

The presented method depends on the residual energy of the nodes and selects cluster heads at random manner. When choosing cluster heads, it is determined whether the present energy of the cluster head exceeds the network’s average total energy in each cycle. If the cluster head has more energy than the sum of all the nodes, it can advance to the next round without losing its position to the other node. However, if the head node has lower energy than the mean node, one of the other nodes is chosen at random manner to be the cluster head after being checked to see if it has the required amount of energy. To become the new cluster head in the r cycle, the head node must send an appropriate signal, which includes data identity. Each cluster head will therefore be able to gather data from cluster nodes and send it to the SDN controller. Algorithm 1 displays the pseudo code for the proposed algorithm of the cluster head selection stage.
**Algorithm 1**: Selection of cluster head:Data input: {(E1), (E2),.., (En)} For each cycle r MeanValue_nodeenergy_ = average(AllNode_energy in network_)    For each cluster c of {(C1), (C2),.., (Cn)}      If HeadNode_energy_ < mean(node_energy_)      choose random node in the cluster c with energy > mean.      End If   End For End For

Each regular node can send the observed data to its allocated cluster head when clusters and cluster heads have been formed. Each cluster head communicates the acquired data to the SDN controller once all data has been received. Algorithm 2 depicts the pseudo-code for the proposed data transfer step in the SDN layer.
**Algorithm 2**: Data transmission step in SDNFor each cluster c of {(C1), (C2),.., (Cn)}    Calculate distance *D* from appropriate cluster nodes;    Transmit data from Cluster heads to the SDN controller; End For Output: {Each cluster node distance *D*, total number of clusters Cn}

### 3.2. Security Enhancement of the Proposed Framework

We integrate emerging technologies, such as SDN and blockchain, and implement various IIoT devices that share data and conduct transactions. In the proposed SDN architecture, the controller provides security and connectivity functions to IoT devices with the help of blockchain technology. The SDN controller implemented to reorganize the data in useful ways and to determine the final location of a given data in the perception layer. The proposed blockchain model monitors the data record and maintains the privacy of the data requested for a transaction. As a result, SDN can identify network irregularities and outsider attacks while excluding harmful packets from its domain. Additionally, it enhances IoT device security and lowers energy use. Furthermore, by preserving the identity of IoT devices in an irreversible public ledger, blockchain offers encryption, security, transparency, authenticity, etc. 

The blockchain guarantees the security of millions of IoT devices through a fully decentralized framework. IIoT devices demand access to the SDN controller, which registers the devices and assigns them an IP address. Even so, the SDN controller continuously monitors all IoT device functional activity and exchanges for security reasons. Furthermore, the SDN controller will disable IIoT devices if any harmful activity is discovered. The IP addresses of IIoT devices are then recorded on decentralized blockchain networks to prohibit them from harmful activities in the clusters.

### 3.3. Distributed Blockchain Model for Smart Industry

The IIoT ecosystem provides security, reliability, privacy, authentication, non-repudiation, and identity management through distributed ledger technology. Along with an open platform, the blockchain includes important elements, such as a decentralized network, transparency, autonomy, immutability, and confidentiality. We have developed a decentralized blockchain-based SDN-IoT model using an energy-aware CHS algorithm. This shared blockchain uses several decentralized controllers. The blockchain is a distributed ledger that lacks independent control or permission. The most recent transactions are entered into the public ledger after being verified by miners. A block is typically released every 5 to 10 min; miners try to produce a scary probabilistic uncertainty based on a cryptographic technique.

The miner blocks have been created with the help of a consensus mechanism. The newly mined block is attached to the blockchain network. The miners continue to add specific blocks to the various blockchain they create. Working on data reconstruction through a block after it has been added to the network is fascinating, mainly because it necessitates turning all remaining blocks. Every specific block that will be paired to the network requires consensus from any significant predominance of nodes across the multiple networks. Many networking systems have covered the distributed Ethereum blockchain network architecture in detail. With the help of this blockchain-enabled proposed architecture, data may be sent from IIoT devices to SDN layers. The data is then forwarded to the smart system for other decision-making processes after being stored in the database. However, the SDN controller first verifies the transaction using specific mining techniques as part of the Ethereum blockchain network architecture before any modifications to the storage are made. After verification, Ethereum will upload the data to a cloud storage service and store the indexes using blocks. The smart industry will be able to communicate with cloud storage through the perception layers in the proposed architecture. The feature of SDN is to check and authenticate the data request information from the perception layer.

## 4. Simulation Setup and Performance Analysis

This section describes the simulation setup and performance assessments of the proposed cluster head section algorithm for blockchain enable SDN-IoT networks. Table 1 describes the comparison of the proposed model with existing methods.

### 4.1. Development of the Blockchain-Enabled SDN-IoT Network’s

We have designed a layered platform that collects data plane records with the help of an SDN controller. The SDN controller allows the data plane only if it gets the proper request. In our proposed approach, we implement the SDN controller using the python language and develop a built-in framework for network management. In addition, we have integrated a blockchain-enabled decentralized ledger that is reachable through a built-in framework. To develop the OpenFlow data plane and distributed models, we use the Mininet-WiFi emulator, in which a python code is implemented and simulated. We developed a framework, layered architecture, which collects the requests from the data plane and updates them to the SDN controller environment. Finally, we debug our emulator with a layered configuration per our proposed blockchain-based SDN integrated IoT network. The distributed ledger we implement is also integrated with a layered framework for the necessary security and privacy in our proposed IoT environment. In each IoT node, we record a block identity, a timestamp, a consensus mechanism, and the hash of the preceding block. 

We set up the experimental platform based on the proposed simulation environments for the performance measurements. As presented earlier, we have implemented the proposed network topologies with the help of Mininet-WiFi for the emulation, Vechain as a blockchain, and a cluster head section for optimizing the SDN controller. All the simulations are executed on general-purpose architecture with Intel processor i5 CPU@2.50 GHz and 8 GB RAM, with Ubuntu as the operating system. Table 2 provides the simulation setup design parameters for the implementation of the proposed architecture.

### 4.2. Proposed Cluster Head Selection Model Result Evaluation

**Figure 4:**
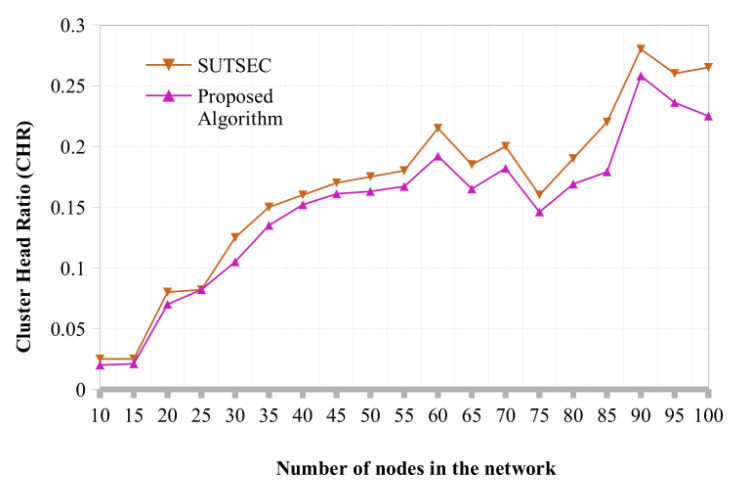
Performance analysis of cluster head selection algorithm.

**Cluster Head Ratio:** The CH ratio concerning the number of IoT nodes is shown in Figure 4. Since CHs have more links, it will be preferable if we choose fewer CHs from the architecture. We developed an algorithm that efficiently selects the configuration with the fewest CHs while also taking an energy-aware approach. We have compared the proposed architecture with the existing model [32] to evaluate the algorithm’s performance. The proposed model and baseline CHS are nearly the same when there are ten IoT nodes. In the case of 25 switch nodes, the proposed algorithm outperformed the baseline and selected fewer cluster heads. Likewise, the baseline chooses fewer CHs for 70 nodes, but the proposed algorithm improves performance as the number of nodes increases beyond 80 nodes.

**Figure 5:**
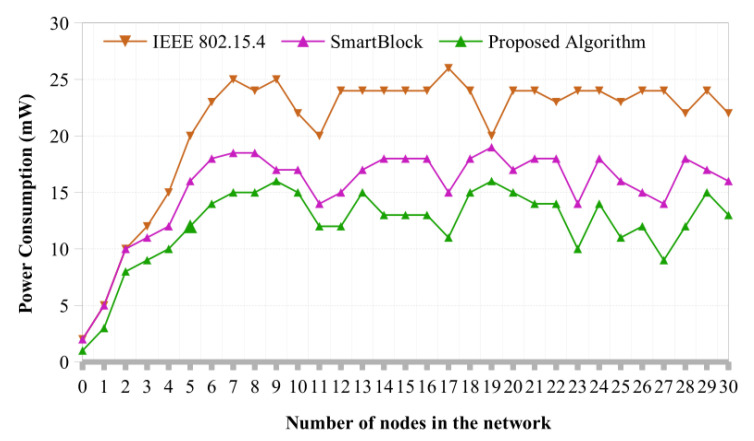
Power consumption of cluster head selection algorithm.

**Power Consumption:** Additionally, Figure 5 displays the power usage (mW) related to the number of nodes. The proposed algorithm outperforms the IEEE 802.15.4 protocol [33] approach protocol in terms of performance. The proposed algorithm performs significantly better than IEEE 802.15.4 protocol for nodes with less than three. Compared to earlier studies, such as the IEEE 802.15.4, it offers a superior outcome for 20 to 25 nodes.

Analyzing an algorithm’s computation cost is also crucial for real-time IoT applications. In general, an algorithm’s input size is crucial. It will be either worst-case or best-case complexity, according to the magnitude of the input. We must choose CHs from the IoT nodes based on our proposed CHS algorithm. We first choose the clusters based on the number of nodes. IoT nodes are distributed equally throughout each cluster. The next step is to identify the nodes with the energy-aware approach to each other. We must compare every node in a cluster to determine which has the highest energy value; if we can locate the sorted list of nodes in the shortest amount of time, which is the best case complexity. However, our major objective is finding the CHs that have the most remaining energy and can gather data and send it to the BS for additional processing. Additionally, the number of additional resources the nodes use affects the network’s overhead. We do not allocate excessive memory for IoT nodes in our proposed approach. 

**Figure 6:**
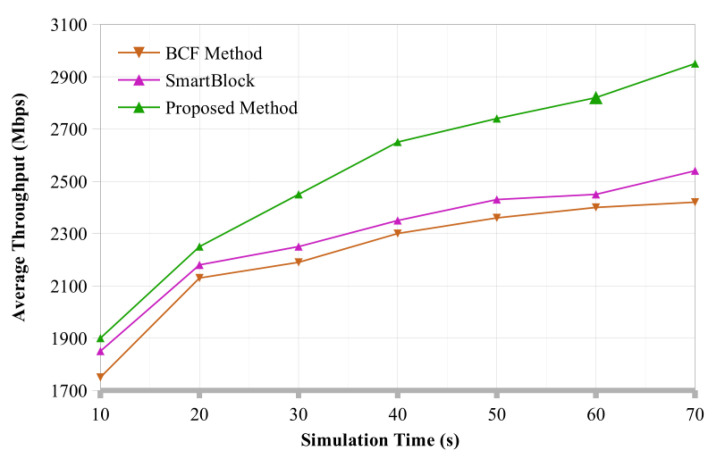
Throughput analysis of proposed system compared with existing methods.

**Overall Throughput:** We examine the proposed model’s throughput, represented in Figure 6 determines how many transactions demands may be transferred from sender to receiver within a suitable time. However, we have considered the architecture, which includes the baseline and existing model [10] for comparisons, and the proposed model has an optimal throughput that begins at 100 transaction demands. When there are 400 queries, all architecture’s capabilities are nearly identical.

Furthermore, the proposed architecture exhibits a much higher throughput than the baseline model when the number of transaction demands reaches 1200. The proposed framework outperforms the existing model regarding throughput for 2400 transactions demand. Moreover, throughput grows as the demand increases, and the proposed architecture performs significantly better on the model than the existing baseline architecture.

**Figure 7:**
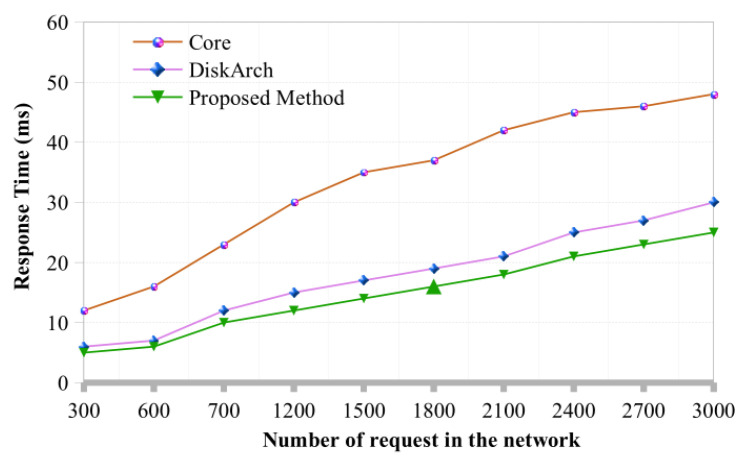
Overall response time of Blockchain enable SDN-IoT architecture.

**Response Time:** Response time is the quantity of time required to receive the system’s initial response. A reduced latent period compared to the existing approach is much more efficient. Figure 7 shows the proposed architecture response time and how it outperforms other existing SDN architectures. The baseline [34] and the proposed architecture require the same amount of time to respond at first when there are only a few hundred transactions. In case more transactions are added to the system, the response time of the baseline model increases. With increased transactions, it is evident that the baseline architecture takes more time to respond to a request with an action. The proposed architecture and distArch [35] operate similarly when the number of requests stays under 1200. However, when the number of requests exceeds 1200, the proposed model responds more quickly than the baseline architecture. The efficiency of the baseline architecture will be worse than that proposed when transaction demand increases to above 1600. However, existing and the proposed architecture function very similarly in the network.

For real-time industrial applications, the time limitations must be considered, and network operating time must be reduced to enhance the system’s performance. Indeed, using innovative technologies, such as blockchain, SDN, and IoT effectively reduces the time required for system integration. Resource management is becoming a difficult task with increased real-time IoT applications. In the proposed work, we have developed an energy-aware blockchain-SDN integrated IoT network for resources management in the framework. Finally, SDN integrated IoT offers the programmability and adaptability needed to manage and regulate industrial activities. 

## 5. Conclusions

Blockchain-based SDN integrated IoT networks struggle with insufficient process specifications in the early stages of development and a lack of resources to implement and operate such a system effectively. However, only a few research efforts have examined and resolved these problems. We proposed and presented a novel energy-aware cluster-head selection model and a decentralized blockchain technique which ensures the network’s continuity and security. We also introduced an enhanced framework for managing the resources in the blockchain-based SDN integrated IoT network. Our SDN framework’s design is established with several homogeneous SDN controllers, which can improve the IoT environment’s scalability, confidentiality, and stability. Our proposed algorithm provides an effective model for choosing the cluster heads with the energy-aware technique, which is necessary for an edge computing environment. Furthermore, the experiment results show that our approach performs better than the conventional model in terms of both energy usage and latency. Compared with a baseline network, the blockchain-enabled SDN-IIoT platform achieves greater efficiency in terms of overall throughput, energy-aware model, and latency. In the future, an access control mechanism will be integrated into SDN framework to enhance the security features of the proposed architecture for industrial IoT application.

## Figures and Tables

**Table 1:** The security performance comparison of different blockchain models.

Authors	Energy Aware Node Selection	Distributed	Node Solidity	Node Optimization	Trustability
Aslam et al. [21]	×	✓	✓	✓	✓
Behera et al. [25]	×	✓	×	✓	×
Farman et al. [31]	×	✓	✓	×	✓
Rahman et al. [24]	✓	✓	×	×	×
Proposed	✓	✓	✓	✓	✓

**Table 2:** Design Parameters of simulation environment.

Design Parameters	Values
**Basic design parameters**	
Network emulator	Mininet-Wifi
Packet analyzer	Wireshark
Storage platform	OpenStack
**SDN Design Parameters**	
SDN routing technique	OpenFlow
No. of SDN controllers	6
**Blockchain Design Parameters**	
Platform	Vechain
Consensus mechanism	Proof of Authority (PoA)
Block size	5 bytes
Block header	85 bytes
**IIoT Design Parameters**	
No. of IoT devices	100
Simulation Time	400 s
Simulation Area	3000 m^2^
Data rate	12 Mbps
IoT energy initial value	11–15 J
Initial trust value	4 J
Transmit node size	0.5–1 MB

## Data Availability

The data sources employed for analysis are presented in the text.
